# Do Dark Humour Users Have Dark Tendencies? Relationships between Dark Humour, the Dark Tetrad, and Online Trolling

**DOI:** 10.3390/bs14060493

**Published:** 2024-06-11

**Authors:** Sophie Voisey, Sonja Heintz

**Affiliations:** School of Psychology, Faculty of Health, University of Plymouth, Drake Circus, Plymouth PL4 8AA, UK

**Keywords:** humour, Dark Tetrad, online trolling, dark personality, dark humour

## Abstract

Humour and antisocial behaviour on the internet are under-researched. Online spaces have opened a gateway for new ways to express unrestrained humour (e.g., dark humour) and ways to behave antisocially (e.g., online trolling). The tendencies and motivations of those engaging with such humour and behaviour are yet to be clearly established and understood. The present study aimed to fill this gap by exploring the interplay between dark humour, online trolling, and dark personality traits. Participants (*N* = 160) completed an online survey consisting of trait scales to assess the Dark Tetrad, dark humour, and online trolling, as well as two online trolling tasks (enjoyment and ability) and two dark humour meme tasks (enjoyment and ability). The results confirmed relationships between the Dark Tetrad and the dark humour trait, and several Dark Tetrad traits were related to the enjoyment of and ability to produce dark humour. Furthermore, dark humour and online trolling were closely related. The findings also revealed that online trolls did not enjoy being trolled but did enjoy trolling, and this ability to troll is underpinned by sadism. These findings illustrate the potential dark psychological motivations for using dark humour, demonstrate that online trolling is infused with darker forms of humour, and provide deeper insights into online trolls.

## 1. Introduction

The prevalence of social media in society has given individuals new ways to express humour and perform antisocial behaviours online. According to a survey conducted in the UK, over 25% of respondents reported being a victim of online trolling at some point [1], and an astounding 75% of 13–36-year-olds have reported sharing memes online [2].

Memes can express a range of humour styles and different humorous topics. Memes refer to pieces of media that are “passed very quickly from one internet user to another, often with slight changes that make it humorous” [3]. Taboo and controversial topics can be taken humorously, often by those unaffected by the topic. Although this “dark” approach to humour has been recorded and used historically (e.g., [4]), recently, this style of humour has taken new formats (e.g., memes). However, not everyone appreciates this dark approach to sensitive topics. This raises the question of why some individuals enjoy dark humour and others do not. Given that dark humour has the potential to offend, and negative humour/comic styles have been linked to dark personality traits in the past (e.g., [5,6,7,8]), it is important to investigate the links between dark personality traits and dark humour to unveil the potential dark motivations behind its use.

Furthermore, an online antisocial behaviour that has recently caught the attention of researchers is online trolling, which is the practice of behaving in a deceptive or disruptive manner on the internet with no apparent instrumental purpose [9]. Trolling has previously been linked with dark personality traits and aggressive humour styles (e.g., [9,10]). These pre-existing associations and conceptual similarities encourage the investigation of the interplay between dark personality traits, dark humour, and online trolling.

### 1.1. The Dark Tetrad

Personality traits refer to an individual’s characteristic patterns and the psychological mechanisms behind them [11]. Traditionally, the five-factor model of personality [12] evaluatively describes relatively neutral traits to categorise personality. However, this model does not account for dark traits we possess. The Dark Triad (e.g., [13]) refers to the three personality traits that construct our dark personality [14]. Initially, sadism had been investigated separately from these traits. However, research has suggested that it should be added alongside the Dark Triad traits (e.g., [15,16]) to create the Dark Tetrad:

1. Machiavellianism is defined by self-interest and tendencies towards deception and manipulation [17], and it is related to externalising behaviours such as dishonesty and cheating [6].
2. Psychopathy can be summarised by high thrill-seeking behaviour and low empathy [18], and it has also been associated with externalising behaviours [19]. Ref. [18] identified a four-factor structure of psychopathy, consisting of callous affect, criminal tendencies, erratic lifestyle, and interpersonal manipulation.
3. Narcissism can be described as self-absorption and feelings of superiority and entitlement, which can also be subdivided into four scales [20], namely, exploitativeness/entitlement, leadership/authority, superiority/arrogance, and self-absorption/self-admiration.
4. Sadism is defined as callousness, deriving pleasure from the suffering of others, and the enjoyment of cruelty [15].

Although these four traits can take extreme clinical forms (e.g., narcissistic personality disorder), in the context of the Dark Tetrad and this study, these traits refer to everyday, subclinical forms. The Short Dark Triad (SD3) [21] is a self-report scale that measures the Dark Triad traits. Due to the addition of sadism, the Short Dark Tetrad (SD4) [22] was developed to measure all four traits but has limited use in the personality literature to date.

The Dark Tetrad traits have been implicated in a range of problematic behaviours (see [23] for a review). Psychopathic and sadistic traits have been associated with online trolling (e.g., [9]). Additionally, positive correlations between negative humour styles and psychopathy and Machiavellianism have been found [6,8]. These two traits have also been associated with darker comic styles [5,7,24], and sadism has been related with the joy of laughing at others (katagelasticism] [7]. Further research has suggested people who score highly on these three traits are more likely to appreciate sexist humour [25]. Associations between these traits and negative humour styles have inspired interest into how they correlate with dark humour, which is yet to be investigated.

### 1.2. Dark Humour

Dark humour treats subjects like death, disease, disability, or warfare with bitter amusement [26,27,28], presenting such morbid and serious topics in a humorous manner [29]. It is closely related to the negative humour styles proposed by Martin et al. (2003) but differs due to the level of social and moral norm transgression and, consequently, the level of surprise experienced [30].

Despite experimental research into dark humour being recent, dark humour is not a newly founded concept. Ref. [4] investigated “gallows humour”, which was used in nations that were oppressed by their invaders. Furthermore, [31] theorised that dark humour was a way for the ego to refuse becoming distressed by reality. More recent research has focussed on dark humour as a coping mechanism. Ref. [32] conducted an analysis of an online forum used by people with cancer; it was used to find relief in their life-threatening situations by joking about embarrassing experiences. Using dark humour to cope has also been noted in emergency service personnel [33,34] and Holocaust survivors [35]. This early research on dark humour focused on jokes told by the victim of the distressing topic.

By contrast, dark humour is often used and enjoyed by people who are not directly impacted by the topic. Due to the internet’s anonymity and new humour forms, dark jokes have undoubtedly become more common and unrestrained. There has been contemporary experimental research conducted into dark humour in the general population. Ref. [30] examined gender differences in the perception of dark humour. Women rated dark humour cartoons as being more incongruous and less funny than men, suggesting that women are more sensitive to the social norm transgression depicted in dark humour. Dark humour preference has also been linked to higher intelligence [28]; participants who scored highly on dark humour preference and comprehension scored higher on intelligence measures and had higher education levels.

This research is limited in terms of both methods and measures. Experimental dark humour research tended to use outdated dark humour cartoons as stimuli. Although reliable, cartoons arguably no longer represent the complexity and depth that dark humour can have in the modern day. This includes the diverse formats dark humour can take (e.g., memes and videos) and the current distressing topics that are joked about (e.g., 9/11, mental health and COVID-19) (e.g., [35,36,37]). There is limited research investigating why dark humour is used by the general population (e.g., for coping) [36], and, as discussed, much personality research is limited to links with other negative styles of humour [5,6].

### 1.3. Online Trolling

Despite the negative psychological outcomes online trolling can have on victims [38], there has been little research conducted on this online antisocial behaviour. Online trolls are “agents of chaos” on the internet who exploit “hot-button issues” [9]. This behaviour differs from cyberbullying in that it is deceptive, inflammatory, and has no direct purpose [9,23]. There is a general agreement that trolling falls into two categories—verbal trolling and behavioural trolling [39]. Verbal trolling can include personal insults (though it is debated whether these should be considered trolling), exclusion tactics, and false ignorance, and it is often characterised by underlying hostility. Behavioural trolling most often takes place within a gaming context, and can include killing or blocking one’s team, spamming, and going away from keyboard [39].

Limited research has investigated the motivations and goals behind trolling. Ref. [39] identified “triggers” from interviews with self-confessed gaming trolls, which were categorised into three main types: social triggers (e.g., poor gameplay), internal triggers (e.g., boredom), and circumstantial triggers (e.g., game chat). They also identified “trolling goals”, which could be categorised into three main types: personal enjoyment, revenge, and thrill-seeking. Other studies also supported the idea that enjoyment is a central motivation for trolls [9] and that trolling may also serve to express overt and relational aggression [40].

Quantitative research has sought to explain individual differences in trolling behaviours through personality approaches and humour-related motivations. Several studies [9,40,41,42] found psychopathy, as well as Machiavellianism and sadism, most strongly predicted online trolling. For instance, [9] concluded that online trolling is an “internet manifestation of everyday sadism” and that the internet is essentially a sadist “playground”. Due to similar findings, [43] constructed the prototypical troll as being male, high in psychopathy and sadism, and low in affective empathy. Dark personality traits, therefore, specifically sadism and psychopathy, underly the motivations for trolling. Ref. [10] suggested that online trolling could also be explained by humorous motivations. Online trolling was correlated with aggressive humour, and katagelasticism was a predictor of online trolling. Ref. [42] also demonstrated that trolling was associated with aggressive and self-defeating humour. However, the humour studied in this research is limited to the four humour styles assessed in the Humour Styles Questionnaire [44]. Other forms of humour, especially dark humour, may also be closely linked to online trolling behaviour.

### 1.4. The Present Study

As discussed, there are clear gaps and issues with current research into dark personality, dark humour, and online trolling, as well as with investigating the interplay between these three variables to unveil the potential dark motivations behind dark humour and online trolling. We preregistered our aims and hypotheses (https://aspredicted.org/kn5j3.pdf, accessed on 30 April 2024), which we derived from conceptual overlaps between the constructs, as well as previous research findings [7,10].

The first aim of this project is to expand the research into dark humour; that is, how the trait, the enjoyment of, and the ability to enact this humour style are linked to dark personality traits, with the following hypotheses:

- Sadism is positively related to dark humour trait, enjoyment, and ability (large effect).
- Psychopathy is positively related to dark humour trait, enjoyment, and ability (medium effect).
- Machiavellianism is positively related to dark humour trait, enjoyment, and ability (small effect)
- Narcissism is positively related to dark humour trait, enjoyment, and ability (small effect).

The second aim of this research is to explore the relationship between the dark humour trait, its enjoyment, and dark humour ability and the online trolling trait, online trolling behaviour, and its enjoyment, with the following hypotheses:

- Dark humour trait is positively related to online trolling trait.
- Dark humour enjoyment is positively related to online trolling enjoyment.
- Dark humour ability is positively related to online trolling ability.

The third aim of the project is to better understand and expand the knowledge into online trolling. The expected potential relationships are as follows:

- Dark Tetrad traits will predict the ability to troll.
- Trolls will be better at trolling than non-trolls.
- Online trolls will perceive trolling attempts more positively than non-trolls.

In addition to these pre-registered hypotheses, we also explored the relationships between dark humour, dark personality, and online trolling and a general humorous temperament (cheerfulness, seriousness, and bad mood) [45,46].

## 2. Materials and Methods

### 2.1. Participants

A total of 165 participants (132 women, 30 men, and 3 other), with an age range of 18–30 years, participated in this study. University of Plymouth students were recruited through the University’s Participation Pool (164 participants) and were given 1 course credit for their participation. Other participants were recruited through an online forum (1 participant) and did not receive a participation reward. Requirements to participate were being aged 18 or over and being fluent in English.

Participants were excluded from data analysis if they (a) always chose the same response option on any of the self-report scales; (b) completed the study too quickly (more than 20 items/minute i.e., < 444 s); and (c) did not pass at least one of two attention check items (e.g., “Choose X on the scale”). Five participants were excluded based on these criteria: 1 participant for (c); 1 participant for (b); 2 participants for both (b) and (c); and 1 participant for not completing any part of the study. Therefore, data from 160 participants (128 women, 29 men, and 3 other) with an age range of 18–30 years (87.5% were 18–20 years old) were included in data analysis. Varying sample sizes for different analyses are noted in the Results section, as some participants abstained from parts of the study or completed less than 80% of a questionnaire or task.

The minimal and optimal sample sizes were determined before data collection using GPower [47]. The minimum sample size calculated was 67 participants, which would allow us to detect large effects with 80% power and 5% error (one-tailed). Obtaining at least 153 participants would detect medium effects with 80% power and 5% error (one-tailed). Our sample was hence large enough to detect medium-sized effects for the hypotheses.

### 2.2. Measures

#### 2.2.1. The Dark Tetrad

The Short Dark Tetrad (SD4) [22] comprises 28 items, assessing the four traits of dark personality with 7 items per trait: narcissism (e.g., “I have some exceptional qualities”; *α* = 0.73); psychopathy (e.g., “I tend to fight against authorities and their rules”; *α* = 0.73); Machiavellianism (e.g., “Flattery is a good way to get people on your side”; *α* = 0.64); and sadism (e.g., “Some people deserve to suffer”; *α* = 0.75). Items were measured on a 5-point Likert Scale ranging from 1 “strongly disagree” to 5 “strongly agree”.

#### 2.2.2. Online Trolling

Online trolling behaviour was measured according to three subcategories: online trolling trait, enjoyment, and ability.

1. Trait: The Global Assessment of Internet Trolling-Revised (GAIT-R) [43] is an 8-item self-report used to assess online trolling, including items such as “I have disrupted people in comment sections of websites” (*α* = 0.78). Items were measured on a 5-point Likert scale ranging from 1 “strongly disagree” to 5 “strongly agree”.
2. Enjoyment: Participants were presented with the definition of online trolling [9] and an online trolling scenario where they were trolled by two players in an online shooter game (receiving condition). They were asked to rate how funny, boring, offensive, and enjoyable (adapted from [48]) they would find being trolled in this way. Each item was measured on a 6-point Likert scale ranging from 1 (e.g., “very unfunny” or “very unenjoyable”) to 6 (e.g., “very funny” or “very enjoyable”). Two open-ended questions were then asked for exploratory purposes: “How would being trolled in this way make you feel, in your own words?” and “If you had the opportunity, would you troll these players back? Why?”
3. Ability: Participants were presented with an online trolling definition [9] and a second online trolling scenario where they encountered the same two players as in the previous scenario, and now could troll them back (sender condition). Participants were instructed to write down ways they could troll these players back effectively (maximum of 15 ways). They were asked to rate how funny, boring, offensive, and enjoyable they would find trolling someone back on the same 6-point Likert scale. An additional open-ended question was asked: “How would trolling someone back make you feel, in your own words?” The detailed task instructions are available on OSF (https://osf.io/qghyk/?view_only=e3b1819e307f403a8991c20a66b26a47, accessed on 30 April 2024).

#### 2.2.3. Dark Humour

Dark humour was measured according to three subcategories: dark humour trait, enjoyment, and ability.

1. Trait: The Dark Humour Scale [49] is an 8-item self-report used to measure the dark humour trait, including items like “I have fun confronting others with macabre and morbid jokes and banter” (*α* = 0.91). Items were measured on a 7-point Likert scale ranging from 1 “strongly disagree” to 7 “strongly agree”.
2. Enjoyment: Dark humour enjoyment was measured through the judgement of memes obtained from the internet. Seven different memes were presented and rated according to how funny, boring, offensive, and enjoyable participants found each one (α = 0.88). Each item was measured on a 6-point Likert scale ranging from 1 (e.g., “very unfunny” or “very unenjoyable”) to 6 (e.g., “very funny” or “very enjoyable”).
3. Ability: Dark humour ability was measured through participants’ ability to produce punchlines for seven blank meme templates obtained from the internet. The definition of dark humour was presented [26,27,29,30], followed by seven different blank meme templates. Participants were asked to think of dark humour punchlines which could fit each template, with a maximum of five punchlines per meme. The resulting punchlines were scored for quantity (i.e., total number of punchlines; *α* = 0.95) and quality. The latter consisted of the authors rating the wittiness (based on the guidelines of [50]) and dark humour (based on the definition of dark humour) of the best punchline per meme from 1 to 5. The authors’ ratings correlated 0.30–0.59 (*p* ≤ 0.001; *Mdn* = 0.43) per meme for wittiness (*α* = 0.80) and 0.59–0.77 (*p* < 0.001; *Mdn* = 0.66) per meme for dark humour (*α* = 0.88). The memes employed in this study are available on OSF (https://osf.io/qghyk/?view_only=e3b1819e307f403a8991c20a66b26a47, accessed on 30 April 2024).

#### 2.2.4. Humorous Temperament

The State-Trat Cheerfulness Inventory Trait Version (STCI-T30) [45,46] assesses trait cheerfulness, seriousness, and bad mood, with 10 items each. Sample items are “I am a merry person” (cheerfulness; *α* = 0.85), “In my life, I like to have everything correct” (seriousness; *α* = 0.78), and “I am often sullen” (bad mood; *α* = 0.87). Each item was measured on a 4-point Likert scale ranging from 1 (“strongly disagree”) to 4 (“strongly agree”).

### 2.3. Procedure

Participation in this study was carried out online using the survey platform Qualtrics. Participants were briefed prior to participation through an information sheet, and they gave their online informed consent. The study began by taking demographic information (for supplementary analyses), followed by the trait scales administrated in a random order. After these, the online trolling tasks and dark humour tasks were presented in a random order. To end, non-student participants were given the opportunity to create a unique participant code should they wish to withdraw their data (students were automatically given a unique SONA code). They were then presented with an online debrief sheet, whereafter, students received 1 course credit. Participants were able to skip past any part of the study they wished not to complete; no sections, apart from consent, were mandatory. The median time taken to complete the study was 33 min. This research was granted full ethical approval from the University of Plymouth Faculty of Health Ethics Committee and complied with the British Psychological Society’s ethical guidelines.

### 2.4. Analysis

The data was analysed via descriptive statistics, Pearson correlations, reliability analyses, and multiple regressions, using R studio [51]. Packages used for analysis and visualisations were Tidyverse [52], BayesFactor [53], Psych [54], and GGally [55]. Results were evaluated via traditional null-hypothesis testing (*p* < 0.05), effect sizes (*r*, using guidelines by [56,57]; |0.10| = small effect; |0.20| = medium effect; |0.30| = large effect), and Bayesian analyses (with *BF* > 3 as evidence for the alternative hypothesis, and *BF* < 0.33 as support for the null hypothesis) [58]. Reliabilities were calculated using Cronbach’s Alpha. The methods and analyses for this research project were preregistered at https://aspredicted.org/kn5j3.pdf (accessed on 30 April 2024). In line with open science practices, we confirm that we have reported all measures, conditions, data exclusions, and how we determined the sample size. The R scripts and data used in this study are available on OSF (https://osf.io/qghyk/?view_only=e3b1819e307f403a8991c20a66b26a47, accessed on 30 April 2024).

## 3. Results

Table 1 provides an overview of the descriptive statistics of the study variables. All variables were approximately normally distributed.

### 3.1. Dark Humour and the Dark Tetrad

Hypothesis 1 explored how the dark humour trait, its enjoyment, and dark humour ability link to the levels of the Dark Tetrad (see Table 2). Sadism was positively correlated with both dark humour trait and enjoyment (large effect), rated wittiness (medium effect), and darkness (large effect), but its relationship with quantity was inconclusive (small effect). Psychopathy was positively correlated with the dark humour trait (large effect). It showed inconclusive relationships to dark humour enjoyment (medium effect), quantity (small effect), wittiness (small effect), and darkness (medium effect). Machiavellianism was positively correlated with the dark humour trait (large effect) and dark humour enjoyment (large effect). The relationship between Machiavellianism and dark humour ability quantity and darkness was inconclusive (small effects), while it was positively related to wittiness (medium effect). Narcissism was positively correlated with the dark humour trait (medium effect), wittiness (medium effect), and darkness (medium effect) but not with dark humour ability (small effect) and dark humour enjoyment (medium effect).

### 3.2. Dark Humour and Online Trolling

Hypothesis 2 investigated the relationships between the dark humour trait, its enjoyment, and dark humour ability and the online trolling trait, online trolling behaviour, and its enjoyment (see Table 3).

Dark humour trait was significantly most strongly positively correlated with the online trolling trait (large effect). It was also positively correlated with online trolling production (large effect), but its relationship with online trolling enjoyment was inconclusive (small effect). Dark humour enjoyment was inconclusively correlated with online trolling enjoyment (small effect). However, it was significantly positively correlated with both the online trolling trait (large effect) and online trolling ability (large effect).

Dark humour ability quantity was most strongly positively correlated with online trolling ability (large effect). Quantity was also positively correlated with the online trolling trait (medium effect) but not with online trolling enjoyment (small effect). Wittiness was positively related to both the online trolling trait (medium effect) and online trolling ability (large effect). Finally, darkness was most strongly related to the online trolling trait (strong effect) and positively related to online trolling enjoyment (medium effect) and online trolling ability (large effect).

### 3.3. Dark Tetrad and Online Trolling

Hypothesis 3 aimed to expand the analysis into online trolling and how it relates to the Dark Tetrad. Out of the Dark Tetrad traits, only sadism predicted the ability to troll (medium effect, *r* = 0.26, *p* = 0.001, *BF* = 32.63). The relationship between Machiavellianism and online trolling ability was inconclusive (small effect, *r* = 0.18, *p* = 0.022, *BF* = 2.33). Psychopathy (no effect, *r* = 0.08, *p* = 0.337, *BF* = 0.29) and narcissism (no effect, *r* = 0.05, *p* = 0.540, *BF* = 0.22) showed evidence for the null hypothesis in relation to online trolling ability. Further, the online trolling trait was positively correlated with online trolling ability (medium effect, *r* = 0.21, *p* = 0.007, *BF* = 6.14). However, the online trolling trait was not correlated with online trolling enjoyment (small effect, *r* = 0.15, *p* = 0.056, *BF* = 1.08).

### 3.4. Supplementary Analyses

Additional analyses were conducted into relationships not previously preregistered or addressed in Hypotheses 1–3 above.

#### 3.4.1. Trolling Enjoyment

After participants wrote down the ways in which they could troll someone back (max. 15 ways), they were asked to rate how funny, boring, offensive, and enjoyable they would find trolling someone back (enjoyment of trolling back).

All the Dark Tetrad traits, except narcissism (*r* = 0.08, *p* = 0.32, *BF* = 0.30), were positively correlated with the enjoyment of trolling back: sadism (*r* = 0.47, *p* < 0.001, *BF* > 100), Machiavellianism (*r* = 0.34, *p* < 0.001, *BF* > 100), and psychopathy (*r* = 0.24, *p* = 0.002, *BF* = 13.48). Enjoyment of trolling someone back was also positively correlated with dark humour enjoyment (*r* = 0.36, *p* < 0.001, *BF* > 100). Enjoyment of trolling someone back was also positively related to the online trolling trait (*r* = 0.44, *p* <.001, *BF* > 100), online trolling enjoyment (*r* = 0.36, *p* <.001, *BF* > 100), and online trolling ability (*r* = 0.20, *p* = 0.010, *BF* = 3.50).

#### 3.4.2. Online trolling and the Dark Tetrad

In line with previous literature, all the Dark Tetrad traits were positively correlated with the online trolling trait (all large effects, *p*s < 0.001, *BF*s > 100). The strongest correlations emerged with sadism (*r* = 0.62), and the weakest correlations with narcissism (*r* = 0.31).

#### 3.4.3. Humorous Temperament, Dark Humour, Online Trolling, and the Dark Tetrad

To determine if the relationships we found for dark humour would also hold for sense of humour more generally, we explored how cheerfulness, seriousness and bad mood, which together form the temperamental basis of humour, relate to dark humour, online trolling and the Dark Tetrad (see Table 4).

Cheerfulness only showed a positive relationship with narcissism (medium effect), while all other relationships with the Dark Tetrad, online trolling, and dark humour were either inconclusive or showed no evidence for a relationship. Seriousness only showed a negative relationship with online trolling enjoyment (medium effect), while all other relationships with the Dark Tetrad, online trolling, and dark humour were either inconclusive or showed no evidence for a relationship. Finally, bad mood showed positive relationships with two of the Dark Tetrad traits, namely, Machiavellianism and sadism (medium effects), as well as with the online trolling trait (medium effect) and the dark humour trait (large effect). All other relationships with online trolling and dark humour were either inconclusive or showed no evidence for a relationship.

## 4. Discussion

The present study explored relationships between dark humour, the Dark Tetrad, and online trolling. As predicted, all the Dark Tetrad traits were positively correlated with the dark humour trait. Sadism and Machiavellianism were positively correlated with dark humour enjoyment. In terms of dark humour ability, sadism, Machiavellianism, and narcissism were positively correlated with wittiness and darkness, while none of the traits predicted dark humour quantity. Furthermore, the dark humour trait was correlated with the online trolling trait, and dark humour ability was correlated with online trolling ability. However, dark humour enjoyment was not positively correlated with perceiving trolling. Online trolls were better at trolling than non-trolls, and sadism was correlated with this ability. However, online trolls did not perceive being trolled more positively than non-trolls.

### 4.1. Hypothesis 1

Hypothesis 1 aimed to investigate the underexplored links between dark humour and the Dark Tetrad. Other negative forms of humour have been linked to psychopathy and Machiavellianism and sadism with katagelasticism [7]. Dark humour presents itself as an extreme form of negative humour (due to the social norm transgression) and incorporates elements of katagelasticism. In line with these observations, these three Dark Tetrad traits, in particular, predicted the dark humour trait; sadism and Machiavellianism predicted dark humour enjoyment; and sadism, Machiavellianism, and narcissism predicted the quality of dark humour production (but not the quantity). The dark humour trait encapsulates an overall picture of dark humour in an individual, which also includes appreciation and ability. Key elements of these personality traits may explain these relationships.

Individuals high in sadism enjoy the suffering of others; thus, sadism is closely related to katagelasticism [7]. Dark humour involves making light of distressing events, whether they are experienced by said individual or not. Therefore, dark humour may be used and enjoyed by individuals high in sadism to gain pleasure from joking of others’ misfortune. Machiavellian individuals tend to use interpersonal manipulation to ensure success. As [8] discussed, these individuals may use aggressive humour to control others and employ personal gain. This may be the same for dark humour; individuals high in Machiavellianism may use and appreciate dark humour due to its potentially manipulative and intimidating nature. Narcissism’s close relationship with the dark humour trait and ability was initially surprising, given its consistent relationships with more positive humour styles (e.g., [6]) and cheerfulness in the present study. However, due to the narcissistic need for superiority and self-absorption, dark humour may be used to feed into these needs through the belittlement of others. Psychopathy is partially characterised by low empathy and a disregard for others’ emotions. Dark humour may therefore be used to simply “laugh at” people, and the impact this has is disregarded. However, psychopathy was unrelated to dark humour ability, showing that high scorers’ dark humour production was not more common or qualitatively better than those with low scores.

Despite these insights, it is important to consider that not everyone will have bad intent when using dark humour. As discussed, many individuals use it to cope with their own personal circumstances. Considering the popularity of dark humour, the intent underlying it may be moderated by further factors not investigated in this study (e.g., political beliefs, katagelasticism, and relationship between humour producer and target).

None of the dark traits predicted the quantity to produce dark humour, but sadism, Machiavellianism, and narcissism were positively related to quality (rated wittiness and/or darkness). This could be due to the clustered responses for quantity, with several participants providing only few punchlines, which is typical for humour production tasks. Ref. [59] reported in their study that only 1/3 of participants wrote down a joke, and 65% were able to write a response to a cartoon. Excuses for not writing down anything included inability to remember jokes, only liking inappropriate jokes, and preferring other genres of humour. In the present study, 92.5% of participants wrote at least one punchline. Although this is impressive, the general cluster around the lower end of responses makes it difficult to establish a correlation between quantity and dark personality traits. By contrast, the quality measure showed the expected relationships for three of the four Dark Tetrad traits (all except psychopathy), with high scorers being wittier and darker in their punchlines than low scorers. Thus, while they did not write down more punchlines, they were able to generate funny responses and to tap into dark content on the spot. This highlights the importance of measuring both the quantity and quality of humour production. To further improve responses, future studies could incorporate a range of production methods, since humour production involves a complex set of skills [59].

The supplementary analyses with humorous temperament showed that dark humour was separate from a general sense of humour as no conclusive relationships emerged between the two concepts (apart from bad mood and dark humour trait). High scorers in Machiavellianism and sadism were characterised by bad mood, while narcissism was underpinned by cheerfulness. The latter finding is in line with previous research relating narcissism to positive humour styles (e.g., [6]). The relationship with bad mood could potentially show that being grumpy, cynical, and sad underlies two of the Dark Tetrad traits as well as general dark humour tendencies, which makes high scorers less able to laugh, engage in humour, and enjoy humour in general. Their humour style seems to tend towards more malicious and mean-spirited forms of humour (sarcasm, ridicule, mockery), including dark humour.

### 4.2. Hypotheses 2 and 3

Hypothesis 2 explored the relationships between dark humour and online trolling due to past research linking this behaviour to other negative styles of humour and katagelasticism [10,42]. Hypothesis 3, overall, aimed to better understand online trolls.

The results indicated that those high in the dark humour trait were also high in the online trolling trait, and those good at producing dark humour (quantity and quality) were also good at producing online trolling attempts. However, in terms of ability, it is important to consider that this correlation may have been influenced by outliers (despite the variables being approximately normal distributed). As discussed, this is a flaw of production tasks more generally, so caution must be taken when interpreting this relationship. Nonetheless, this confirms that online trolling can also be underpinned by darker forms of humour. However, contrary to predictions, dark humour enjoyment was not most strongly correlated with perceiving being trolled positively (i.e., those who enjoy dark humour do not necessarily enjoy being trolled). This could be because being trolled might not be funny for the victim.

Interestingly, however, supplementary analyses revealed that dark humour enjoyment was positively correlated with the enjoyment of trolling back. That is, those who enjoy dark humour also would enjoy trolling someone back. The act of trolling undoubtedly has more humorous underpinnings than being trolled, as both share banter, irony, and teasing. Clearly, individuals struggle to see the humorous side of being trolled but not of trolling back, indicating there is a disconnect between the two sides of trolling. Even those who scored highly on the online trolling trait did not necessarily score highly on online trolling enjoyment; that is, online trolls do not enjoy being trolled either, but they do enjoy trolling back. This is despite the finding that as online trolling enjoyment increases, so does the enjoyment of trolling someone back. This illustrates that online trolls can “give” but not “take” and further demonstrates that there is a disconnect between the two trolling experiences.

Additionally, only sadism predicted the ability to troll. Clearly, gaining pleasure from the suffering of others is a requirement to not only engage in trolling (e.g., [9]) but also to be good at producing ways of trolling. Moreover, those who scored highly for the online trolling trait were better at producing trolling attempts (i.e., trolls are better at trolling than non-trolls).

Supplementary analyses with humorous temperament showed that the relationship to online trolling is specific to dark humour, as humorous temperament was only weakly related to online trolling overall. We found that enjoyment of trolling was related to lower seriousness, so the more playful a person, the more they would enjoy online trolling attempts. The online trolling trait was positively related to bad mood, which—similar to the Dark Tetrad traits—might indicate that bitterness, sadness, and grumpiness underlies the tendency to troll online.

### 4.3. General Limitations and Future Research

One consideration to make is that participants may have repeated meme punchlines they have seen before, given that the templates were from the internet. This was noticeable when rating the punchlines, as they were often similar or identical across participants. However, even if some participants did repeat dark jokes they had seen before, this is still informative in that they must have some kind of interest to view, retain, and repeat such material. The positive relationship of the different dark humour measures (trait, enjoyment, and the three ability ratings) further supports the convergent validity of these ratings. It should still be kept in mind, however that the dark humour ability tasks tapped more into reproduction than creation, which will likely be the case when these tasks are conducted online where memes and punchlines can be searched.

Furthermore, the sample was predominantly young females. Although this may limit generalisability across the population, the sample recruited is the most relevant for investigating online behaviours; an older sample would not be as familiar with meme-based humour and online trolling. As men are more likely to engage in darker forms of humour and tend to score higher in dark traits (see [60] for a review), recruiting a larger sample of men for robust gender comparisons will be necessary in future studies.

In addition to rating the meme punchlines, it would also be worthwhile to analyse the online trolling attempts according to how good the attempts to troll actually are (i.e., the quality and not just the quantity of online trolling attempts). Additionally, examining the open responses may allow us deeper insights into trolling and being trolled.

Future research may benefit from investigating the role of katagelasticism in the appreciation of dark humour due to its links to both sadism and online trolling [7,10], as well as from relating other humour and comic styles [24,44] to online trolling. Another promising approach to studying the relationship between humour and trolling is integrating pragmatic and linguistic frameworks and concepts such as the mode-adoption of humour performance [61], joking threads [62], roasting [63], and the common core of untruthfulness [64].

## 5. Conclusions

This multimethod study contains the first investigations into how dark humour relates to dark personality and online trolling, incorporating relevant humour appreciation and production tasks. Findings indicated that the Dark Tetrad traits, particularly sadism and Machiavellianism, were related to certain elements of dark humour. This illustrates the potential dark psychological motivations behind the appreciation and use of dark humour. Moreover, online trolling appeared to be underpinned by certain aspects of dark humour. This research also allowed for deeper insights into online trolls. Interestingly, online trolls did not appear to enjoy being trolled, but they enjoy trolling, highlighting a disconnect between the two experiences. Overall, these results indicate that dark humour may be fuelled by dark motivations (e.g., sadistic/Machiavellian ones) and that online trolling is related to darker forms of humour.

## Figures and Tables

**Table 1:** Means, standard deviations, and range of the variables in the study.

Variables	*M*	*SD*	Range	*N*
Dark Tetrad				
Psychopathy	14.82	4.40	7–28	160
Machiavellianism	23.77	3.92	9–32	160
Narcissism	19.35	4.43	7–32	160
Sadism	18.79	5.27	8–34	160
Online Trolling				
Trait	15.30	5.36	8–32	160
Enjoyment	11.69	3.93	4–24	159
Ability	3.46	2.17	0–15	160
Dark humour				
Trait	35.22	10.86	8–56	160
Enjoyment	111.13	23.89	28–167	158
Ability quantity	10.32	9.32	0–35	160
Ability wittiness	2.10	0.56	1.00–4.00	140
Ability darkness	2.39	0.67	1.08–4.21	140
Humour temperament				
Cheerfulness	27.00	4.22	17–36	160
Seriousness	26.51	4.82	14–39	160
Bad mood	21.84	5.88	10–39	160

**Table 2:** Correlations between the Dark Tetrad traits and dark humour measures for Hypothesis 1.

Dark Humour	Psychopathy			Machiavellianism			Narcissism			Sadism		
	*r*	*p*	*BF*	*r*	*p*	*BF*	*r*	*p*	*BF*	*r*	*p*	*BF*
Trait	**0.40**	<0.001	>100	**0.53**	<0.001	>100	**0.25**	<0.001	21.64	**0.56**	<0.001	>100
Enjoyment	0.18	0.010	2.40	**0.29**	<0.001	>100	0.19	0.020	2.85	**0.36**	<0.001	>100
Ability quantity	0.14	0.090	0.80	0.18	0.030	2.11	0.11	0.200	0.47	0.17	0.060	1.52
Ability wittiness	0.17	0.050	1.31	**0.28**	<0.001	45.06	**0.25**	<0.001	12.28	**0.23**	<0.001	8.24
Ability darkness	0.20	0.010	2.69	0.19	0.020	2.52	**0.27**	<0.001	34.70	**0.34**	<0.001	>100

Notes: *N* = 140–160; *BF* = Bayes factor. Correlations with *p* < 0.05 and *BF* > 3 highlighted in bold.

**Table 3:** Correlations between the online trolling and dark humour measures for Hypothesis 2.

Dark Humour	Online Trolling Trait			Online Trolling Enjoyment			Online Trolling Ability		
	*r*	*p*	*BF*	*r*	*p*	*BF*	*r*	*p*	*BF*
Trait	**0.57**	<0.001	>100	0.17	0.020	1.60	**0.34**	<0.001	>100
Enjoyment	**0.32**	<0.001	>100	0.14	0.060	0.82	**0.34**	<0.001	>100
Ability quantity	**0.22**	0.010	9.29	0.15	0.150	1.10	**0.43**	<0.001	>100
Ability wittiness	**0.22**	0.020	4.81	0.20	0.020	2.57	**0.32**	<0.001	>100
Ability darkness	**0.38**	<0.001	>100	**0.23**	0.010	6.04	**0.34**	<0.001	>100

Notes: *N* = 140–160; *BF* = Bayes factor. Correlations with *p* < 0.05 and *BF* > 3 highlighted in bold.

**Table 4:** Correlations between humorous temperament and the Dark Tetrad, online trolling, and dark humour.

Variable	Cheerfulness			Seriousness			Bad Mood		
	*r*	*p*	*BF*	*r*	*p*	*BF*	*r*	*p*	*BF*
Dark Tetrad									
Psychopathy	−0.03	0.724	0.19	−0.13	0.104	0.66	0.17	0.035	1.58
Machiavellianism	−0.10	0.191	0.42	−0.01	0.882	0.19	**0.22**	0.006	7.62
Narcissism	**0.25**	0.001	28.39	0.13	0.103	0.66	−0.11	0.171	0.45
Sadism	−0.16	0.039	1.44	−0.18	0.020	2.58	**0.25**	0.001	29.51
Online trolling									
Trait	−0.10	0.227	0.37	−0.11	0.185	0.43	**0.22**	0.005	8.71
Enjoyment	−0.12	0.150	0.50	**−0.27**	<0.001	54.92	0.10	0.200	0.41
Ability	−0.04	0.580	0.21	−0.10	0.219	0.38	−0.03	0.673	0.20
Dark humour									
Trait	−0.16	0.047	1.22	−0.17	0.027	1.95	**0.32**	<0.001	>100
Enjoyment	0.03	0.733	0.19	−0.12	0.123	0.58	0.07	0.376	0.27
Ability quantity	−0.01	0.863	0.19	−0.10	0.204	0.40	0.03	0.725	0.19
Ability wittiness	0.04	0.628	0.22	−0.14	0.100	0.73	−0.12	0.150	0.53
Ability darkness	0.01	0.883	0.20	−0.17	0.050	1.23	−0.03	0.753	0.21

Notes. *N* = 140–160; *BF* = Bayes factor. Correlations with *p* < 0.05 and *BF* > 3 highlighted in bold.

## Data Availability

The materials and data of the study are available on OSF: https://osf.io/qghyk/?view_only=e3b1819e307f403a8991c20a66b26a47 (accessed on 30 April 2024).
